# Biology, pathotype, and virulence of *Globodera rostochiensis* populations from Kenya

**DOI:** 10.21307/jofnem-2021-003

**Published:** 2021-02-15

**Authors:** James M. Mwangi, Grace N. Mwangi, Maria R. Finckh, Sebastian Kiewnick

**Affiliations:** 1 Chuka University, P.O. Box 109-60400, Chuka, Kenya; 2 International Master of Science in Agro- and Environmental Nematology, Nematology Research Unit, Faculty of Sciences, Ghent University, K. L. Ledeganckstraat 35, 9000, Ghent, Belgium; 3 Kassel University, Nordbahnhofstr. 1a, 37213, Witzenhausen, Germany; 4 Julius Kühn-Institut, Federal Research Centre for Cultivated Plants, Institute for Plant Protection in Field Crops and Grassland, Messeweg 11/12, 38104, Braunschweig, Germany

**Keywords:** Diapause, *Globodera rostochiensis*, *G. pallida*, Management, Pathotype, Resistance genes, Virulence

## Abstract

The potato cyst nematodes (PCN), *Globodera rostochiensis* (Woll.) and *G. pallida* (Stone), are important pests of potato globally. Due to their extensive damage potential and the challenge of managing them, these nematodes are under strict regulations in many countries; however, despite these regulations, PCN continue to spread into new areas and countries. In Kenya, *G. rostochiensis* was first reported in 2015 and *G. pallida* was reported three years later, both in Nyandarua County. Research was conducted to characterize the biology, pathotype, and virulence of *G. rostochiensis* populations from Kenya in glasshouse and laboratory studies. The development of *G. rostochiensis* was assessed in roots of susceptible potato ‘Désirée’ and resistant ‘Laura’ carrying the H1 resistance gene. The ‘HAR1’ population from Kenya and ‘Ecosse’ from Germany were not able to produce females in the roots of the resistant potato ‘Laura’. The rate of root penetration by *G. rostochiensis* juveniles did not differ (*p* > 0.05) between populations and cultivars. However, in the resistant cultivar, juveniles developed into males only. A total of 736 cumulative degree-days at 6°C base temperature (DD_6_) were required by ‘HAR1’ to complete the life cycle on ‘Désirée’, whereas ‘Ecosse’ completed the life cycle within 645 DD_6_. The Kenyan populations lacked obligatory diapause and high numbers of juveniles hatched immediately after maturity. Consequently, the Kenyan populations had the potential to complete up to three reproduction cycles in less than a year. On selected potato cultivars, the populations from Kenya failed to reproduce on 10 out of 13 commercial cultivars tested. The 10 cultivars carried the H1 resistance gene, which suggests that the *G. rostochiensis* populations tested belong to the Ro1/4 pathotype group. The virulence of the *G. rostochiensis* populations from Kenya did not differ from that of the standard reference population ‘Ecosse’ and therefore can be effectively managed with the commercially available potato cultivars carrying the H1 resistance gene.

Potato, *Solanum tuberosum* L., is the second most important food crop in Kenya after maize. It is also ranked third globally in terms of human consumption (Birch et al., 2012). Potatoes in Kenya are mainly produced by small holder farmers owning, on average, less than 2 ha (Gildemacher et al., 2011; Janssens et al., 2013). With potato yields less than 10 metric tons per hectare, the productivity is far below the production potential of >40 metric tons per hectare reported among the leading potato producers (Janssens et al., 2013; FAO, 2019). The low productivity is mostly attributed to biotic and abiotic stressors, key among them, pests, and diseases (Were et al., 2013). Bacterial wilt, caused by *Ralstonia solanacearum,* and late blight, caused by *Phytophthora infestans*, are the major potato diseases in Kenya (Muthoni et al., 2013). Potato pests also include insects such as aphids, white flies, potato tuber moth and millipedes. Plant-parasitic nematodes (PPN) are regarded minor pests of potato in the country, although they attack a wide range of agricultural crops and consequently constrain an already erratic global food security (Jones et al., 2013). Plant-parasitic nematodes often go undetected because symptoms associated with their infestations resemble those caused by other biotic and abiotic factors (Mulder et al., 1997). Until 2015, root-knot nematodes (*Meloidogyne* spp., RKN) were the only economically important PPN associated with potato in Kenya (Were et al., 2013). In 2015, *Globodera rostochiensis* (Woll.) was reported for the first time in Nyandarua County, Kenya (Mwangi et al., 2015). A nationwide survey conducted in 2017 discovered more infested areas and, in addition, *Globodera pallida* (Stone) was discovered in one potato field in Nyandarua County (Mburu et al., 2018, 2020).

The PCN, *G. rostochiensis* and *G. pallida*, are the most damaging nematode pests of potato (Jones et al., 2013). Their status as quarantine organisms (Hockland et al., 2012; EPPO, 2017) has negatively impacted world trade of potatoes (Faggian et al., 2012). The origin of PCN is the Andean region of South America (Mai, 1977; Grenier et al., 2010). PCN later spread to countries where potatoes are grown mainly through movement of contaminated potato materials, contaminated soil, and movement of farm machinery (Mai, 1977; Brodie, 1993; Goeminne et al., 2011). Today, PCN are present in many countries around the world. In Africa, PCN have been reported in South Africa (Knoetze et al., 2006), Algeria (Mezerket et al., 2018), Morocco and Tunisia (Greco, 1993), Kenya (Mwangi et al., 2015; Mburu et al., 2018), Rwanda (Niragire et al., 2019), and recently in Uganda (Cortada et al., 2020).

Potato production in Kenya, and many countries in Africa, is mainly done by small holder farmers whose production is limited by small acreage and lack of resources for input (Janssens et al., 2013). The intensive production is mainly for subsistence use and the excess is sold in the local market (MOALF, 2016). In Kenya, potatoes are grown throughout the year with minimal crop rotation (Muthoni et al., 2013). The majority of farmers do not have access to certified seed potatoes and therefore produce their own seed or source them from the neighborhood (Gildemacher et al., 2011; Mburu et al., 2020). Such practices allow PPN to spread and accumulate to damaging levels.

The presence of PCN in Kenya is a drawback in the effort to upscale food production. Quick interventions are required in order to prevent further invasion and to reduce the nematode densities to manageable levels. Farmers need to adapt an integrated management approach in order to lower the nematode population. The approach integrates the use of proper crop rotation cycles using host and non-host crops (Evans, 1993), use of trap crops (Dias et al., 2012), application of biological control products (Davis et al., 2018), and use of resistant potato cultivars (Molinari, 2011). Chemical application as a management option is expensive and highly restricted due to the adverse effects chemicals have on humans and the environment (Greco, 1993; Sheridan et al., 2004; Faggian et al., 2012). Use of resistant potato cultivars in PPN management is easy for farmers to apply and is environmentally preferable (Molinari, 2011; Davies and Elling, 2015). Cultivars with complete resistance to *G. rostochiensis* are available to the growers. These cultivars contain major resistance (R) genes such as GroVI and H1, which confer resistance to *G. rostochiensis* (Bakker et al., 2006; Dalamu et al., 2012).

For effective utilization of integrated management of PCN, information regarding the population(s) present in the field is needed (Greco, 1993). This includes information on the biology, the pathotype(s), and the virulence of the populations to commercially available resistance genes. This study was therefore undertaken to: (i) study the development of the *G. rostochiensis* ‘HAR1’ population from Kenya in the roots of resistant and susceptible potato cultivars in comparison to the European reference *G. rostochiensis* population ‘Ecosse’ representing the Ro1 pathotype (EPPO, 2006); (ii) investigate if obligatory diapause is present in Kenyan *G. rostochiensis* populations; (iii) determine the pathotype(s) of *G. rostochiensis* populations from Kenya; and (iv) assess the virulence of Kenyan populations on selected potato cultivars in comparison to the reference *G. rostochiensis* population, ‘Ecosse’.

## Materials and methods

### Nematode populations

Seven populations of *G. rostochiensis* were used in this study, six populations were from Kenya and one from Germany. Five of the Kenyan populations, ‘HAR1’, ‘HAR2’, ‘RIR’, ‘KIN1’, and KIN2, were collected from potato fields in Nyandarua County, while the sixth population (‘TGN’) was from Kiambu County. The population *G. rostochiensis* population ‘Ecosse’ is the official reference population for testing potato genotypes for resistance to *G. rostochiensis* pathotype Ro1 (EPPO, 2006). This reference population was obtained from the nematode laboratory at the Julius-kühn Institut (Braunschweig-Germany), where this study was conducted under strict quarantine conditions. The *G. rostochiensis* populations were multiplied twice on the susceptible potato cv. ‘Désirée’ prior to the study. Except for the cysts used in experiments testing the presence of diapause, the remaining cysts were stored at 4°C for at least six months to break the diapause before they were used in this study.

### Planting materials

Potatoes derived from tubers and in vitro propagation were used in the study. The potato cultivars included in the study were; ‘Désirée’, ‘Laura’, ‘Albatros’, ‘Amado’, ‘Seresta’, ‘Papageno’ ‘Belana’, and ‘Ribera’ all derived from tuber. ‘Connect’, ‘Caruso’, ‘Amanda’, ‘Performer’, and ‘Rossini’ were in vitro propagated. For the second set of cultivars, in vitro plants of ‘Désirée’ served as the control. In addition, six differential potato clones; *S. tuberosum* cv. ‘Désirée’, *S. tuberosum* cv. ‘Laura’, *S. kurtzianum* 60.21.19, *S. vernei* 58.1642.4, *S. vernei* 62.33.3, and *S. vernei* 65.346.19 (Kort et al., 1977) were used to test the pathotype(s) of the Kenyan populations.

### Growth substrate and glasshouse conditions

Experiments were conducted in unsterile loess soil (Müller and Rumpenhorst, 2000; Mwangi et al., 2019a). Soil amendment was done at the beginning of the experiment by mixing it with slow release fertiliser (Osmocote Exact Standard^®^ with 15% N, 9% P_2_O_5_, 12% K_2_O and 2% MgO) at a rate of 1.5 g per kg of soil. The glasshouse temperature was set at 18 ± 2°C and relative humidity maintained at a range of 50 to 70% throughout the experiments. Soil and air temperatures were recorded hourly using Testo^®^ 175T3 (Testo Ltd, UK) temperature loggers. Supplementary light was provided during winter to ensure a minimum of 12 hr light per day.

### Preparation of inoculum

Plants were inoculated either with full cysts packed in retrievable bags made up of a 100 µm diameter nylon mesh or with an aqueous suspension of eggs and juveniles. To prepare the inoculum, a known number of cysts were soaked in tap water overnight and then crushed to free eggs and juveniles (Seinhorst and Den-ouden, 1966). A suspension of eggs and juveniles was prepared and the number adjusted to approx. 500 eggs and J2 per ml of water. For inoculation, two, 30 mm deep holes per pot were made in the soil using a plastic rod and nematodes dispensed equally into the holes using a pipette to achieve the desired initial population density (Pi) of five eggs and J2 per ml soil.

### Sample processing

Apart from the experiment on the study of nematode biology, all other experiments were terminated after attaining 1,000 degree-days at 6°C base temperature (DD_6_). This was approx. 12 weeks after inoculation. Cysts were extracted from the entire soil volume by washing the loess through a 250 µl sieve (Mwangi et al., 2019a). The cysts together with plant debris were collected on a filter paper (Macherey-Nagel GmBh and Co. KG) and left to dry at room temperature for a period of two weeks. Cysts were then separated from the debris using the acetone extraction method (Seinhorst, 1974). In all experiments, except the one testing the diapause of *G. rostochiensis*, recovered cysts were counted and then crushed (Seinhorst and Den-Ouden, 1966) to estimate the final number of eggs and juveniles per treatment (Mwangi et al., 2019a). Every experiment was conducted twice under similar conditions.

### Study of *G. rostochiensis* biology

The development of the *G. rostochiensis* population ‘HAR1’ from Kenya in the roots of the resistant potato ‘Laura’ and susceptible potato ‘Désirée’ was assessed in the glasshouse experiments and compared with the reference *G. rostochiensis* population ‘Ecosse’. Eye-plugs were scooped from the pre-germinated tubers of the two cultivars and planted in 192 ml pots. For each of the cultivar-population combinations, 50 potatoes were planted and immediately inoculated with 10 cysts per pot. In the first experiment ‘Ecosse’ cysts were sieved using 500 µm sieve prior to inoculation. However, due to lack of enough inoculum for ‘HAR1’ the cysts used varied in size. In the second experiment, only cysts larger than 500 µm were used for both, ‘Ecosse’ and ‘HAR1’.

The cysts were packed in small retrievable bags made up of a nylon mesh and placed next to the eye-plug. Pots were randomized in the support boxes (Mwangi et al., 2019a) and transferred to the glasshouse benches. Plants were watered as required throughout the duration of the experiment.

To assess the development of the nematodes in the roots, four pots per treatment were randomly selected at 14, 18, 23, 28, 35, 42, 49, 56, 63, and 70 days after planting. Soil adhering to the roots was collected into centrifugation tubes and second stage juveniles (J2s), males and females extracted using the centrifugation flotation method (EPPO, 2013). Roots were then washed and stained in acid fuchsin (Byrd et al., 1983). The stained roots were cut into approx. 10 mm long sections and macerated in water using an ULTRATURRAX^®^ homogenizer (IKA^®^-Werke GmbH and Co. KG) at 1 × 10^5^ rpm for approx. 30 sec. By this, nematodes at different developmental stages were freed from the root tissues into the water to form a suspension. The developmental stages of the nematodes were counted from the entire suspension using a 15 ml counting dish under a Nikon^®^ SMZ1270 stereo microscope. In total, 12 weeks after planting, the experiment was stopped and the final dry cysts extracted from four pots per treatment as described above.

### Investigating the presence of diapause in Kenya *G. rostochiensis* populations

The presence of diapause in the six Kenyan *G. rostochiensis* populations was investigated using two methods. First, the populations were reproduced twice on the susceptible potato ‘Désirée’. After the first reproduction the cysts were stored to break the diapause as described above. The freshly developed cysts from the second reproduction cycle were used in this experiment without storage to break the diapause. In total, 10 fresh cysts (1st generation cysts), for each of the six populations, were packed in small retrievable nylon bags described above with five replications per treatment and buried in the pot next to the tuber of the susceptible cultivar ‘Désirée’. Pots were filled with soil, randomized in the glasshouse, and watered as required. After 12 weeks, the experiment was terminated and cysts extracted and enumerated as described above. The newly extracted cysts were then immediately used for the next inoculation cycle as described above. The experiment was repeated until four generations were completed without diapause.

In a second experiment, the *G. rostochiensis* population ‘HAR1’ was used to test the ability of the newly formed cysts to hatch without diapause. Hatching tests were done in potato root diffusate (PRD) and tap water was used as the control. The PRD was obtained as described by Rawsthorne and Brodie (1986). In total, 20 newly formed cysts (1st generation cysts) were placed in hatching tubes (Mwangi et al., 2019b) with four replications per treatment and hatching media (PRD or water) added. The experiment was left in the dark at room temperature for 10 weeks. Hatched juveniles were counted and hatching solution replaced once per week. At the end of the experiment, the cysts were crushed to determine the number of the unhatched eggs. The hatching tests were repeated using the 2nd and 3rd generation cysts without diapause.

### Testing the pathotype of *G. rostochiensis* from Kenya

Due to limited availability of planting material from differential potato clones, only the pathotype of ‘HAR1’ *G. rostochiensis* population was tested twice in glasshouse experiments on six differential clones (Kort et al., 1977). Pre-germinated tubers of the differential potato clones were planted into 1 L pots with five replications each. In total, 14 days after planting, each pot was inoculated with 5,000 eggs and juveniles of *G. rostochiensis* ‘HAR1’ to achieve a Pi of five eggs and J2 per ml soil. As control, plants were inoculated with the Ro1 reference population *G. rostochiensis* ‘Ecosse’. The pots were left in the glasshouse for 12 weeks and the reproduction factor assessed at the end of the experiment.

### Virulence assessment of *G. rostochiensis* from Kenya

Two different experiments were done to assess the virulence of Kenyan *G. rostochiensis* populations. In the first experiment, the virulence of three *G. rostochiensis* populations (‘HAR2’, ‘KIN1’, and ‘TGN’) from Kenya and the European *G. rostochiensis* population ‘Ecosse’ was assessed on six in vitro derived potato cultivars. At planting, the TC plants used in the study were approx. three weeks old and 30 mm tall. Plantlets of ‘Désirée’, ‘Amanda’, ‘Performer’, ‘Caruso’ and ‘Connect’, and ‘Rossini’ were removed from the growing media and the roots washed in running water followed by planting into 192 ml pots with 10 replications each. The pots were randomized in support boxes and placed on the glasshouse bench. Due to the lack of TC plants, ‘Laura’ was tested using eye-plugs in 192 ml pots. In total, 14 days after planting, each plant was inoculated with 1,000 eggs and J2s of each of the four nematode populations to achieve an estimated Pi of five eggs and J2s per ml soil.

In the second experiment, the virulence of the ‘HAR1’ *G. rostochiensis* population from Kenya was tested on seven tuber derived potato cultivars. The reference *G. rostochiensis* population ‘Ecosse’ was also used for comparison. Tubers of the ‘Désirée’, ‘Albatros’, ‘Seresta’, ‘Papageno’ ‘Belana’, ‘Ribera’, and ‘Amado’ were planted into 1 l pots with five replications. In total, 14 days after planting, each pot was inoculated with 5,000 eggs and juveniles to achieve a Pi of five eggs and J2 per ml soil. The experiment was terminated 84 days after inoculation (DAI) and the cysts extracted. Cysts were then enumerated and crushed to estimate the final nematode population density.

### Data analysis

The cumulative degree-days (DD) were determined by calculating the average daily temperature, minus the base temperature of 6°C (Mugniery, 1978). The reproduction factor (Rf) was calculated by dividing the final nematode density (Pf) by the initial population (Pi). The relative susceptibility (Rs) as percentage of a cultivar was determined using the formula Rs = Pf_test cultivar_/Pf_susceptible control_ × 100, where susceptible cv. ‘Désirée’ was used as the reference cultivar. The resistance level was then rated using the standard scoring notation where Rs < 1% = 9; 1.1-3% = 8; 3.1-5% = 7; 5.1-10% = 6; 10.1-15% = 5; 15.1-25% = 4; 25.1-50% = 3; 50.1-100% = 2; >100% = 1, where 1 is the most susceptible and 9 the most resistant (EPPO, 2006). Mean numbers of nematodes and cysts recovered in each of the experiments were tested for normality and homogeneity of variance using the Shapiro test and Levene’s test, respectively. As there was no significant difference (*p* > 0.05) between the repeated experiments testing diapause and testing virulence with tubers, data were pooled prior to the analysis of variance. Data generated from the study on biology and diapause were analyzed using ANOVA. Means that were significantly different (*p* ≤ 0.05) were separated using Tukey’s HSD test. Kruskal–Wallis test was used to analyze data on virulence assessed on in vitro potato plants. Treatments that were significantly different (*p* ≤ 0.05) were separated using Kruskal post hoc test. Statistical analysis was done with statistical software R (version 3.6.0).

## Results

### Study of *G. rostochiensis* biology

**Figure 1:**
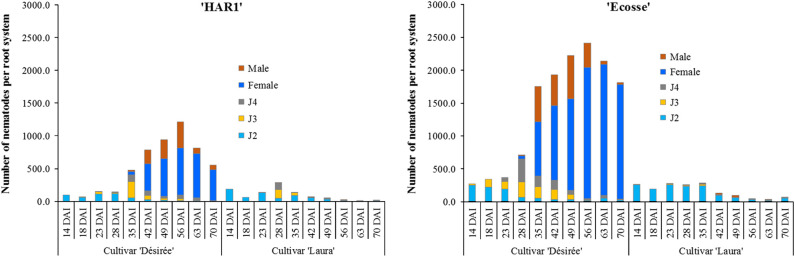
Figure showing the mean number of different developmental stages of *Globodera rostochiensis*, ‘HAR1’ and ‘Ecosse’ populations in resistant cv. ‘Laura’ and susceptible cv. ‘Désirée’ in the glasshouse during the first experiment.

In the first experiment, *G. rostochiensis* J2 were found in the roots of potato for all treatments at 14 days after inoculation (DAI), at 171 DD_6_. The mean number recorded did not differ (*p* > 0.05) among cultivars and populations (Fig. 1). A few third-stage juveniles (J3) of *G. rostochiensis* ‘Ecosse’ were also detected in ‘Désirée’ 14 DAI. *G. rostochiensis* ‘HAR1’ J3 were detected in the roots of ‘Désirée’ 18 DAI (237 DD_6_). At this time, the number of *G*. *rostochiensis* ‘Ecosse’ J3 was 36.3% higher in ‘Désirée’ treatment compared to the same cultivar inoculated with *G. rostochiensis* ‘HAR1’. The number of *G*. *rostochiensis* J2 in the roots varied among treatments (*p* < 0.05) with higher numbers being recorded on ‘Désirée’ (220.0 J3 per root system) and ‘Laura’ (192.3 J3 per root system) inoculated with *G. rostochiensis* ‘Ecosse’ compared to *G. rostochiensis* ‘HAR1’. The first J3 in the roots of the resistant cultivar were detected 23 DAI (302 DD_6_). Overall, the number of J3 was higher in the susceptible cultivar inoculated with *G. rostochiensis* ‘Ecosse’ compared to the rest of the treatments. At 23 DAI fourth-stage juveniles (J4s) were detected in all treatments except for *G. rostochiensis* ‘HARI’ on ‘Laura’ which appeared on 28 DAI. In total, 28 days and 367 DD_6_ were required for the first males and females to emerge from ‘Désirée’. There were 62% more females in ‘Désirée’ inoculated with *G. rostochiensis* ‘Ecosse’ than with ‘HAR1’. From 35 DAI (463 DD_6_), males were detected in the roots of ‘Laura’, for both *G. rostochiensis* populations, until 63 DAI (840 DD_6_) when the numbers diminished. The number of females extracted from the roots increased steadily from 35 DAI (463 DD_6_) with more *G. rostochiensis* ‘Ecosse’ females counted on the susceptible cultivar compared to *G. rostochiensis* ‘HAR1’ throughout the experiment. However, no females were detected in resistant ‘Laura’ for either populations (Fig. 1). The first brown cyst of *G. rostochiensis* ‘Ecosse’ was isolated from the soil 49 DAI (646 DD_6_) and for *G. rostochiensis* ‘HAR1’ seven days later at 741 DD_6._ The mean numbers of cysts at the end of the experiment were 918 and 705 cysts per root system for *G. rostochiensis* ‘Ecosse’ and ‘HAR1’, respectively.

**Figure 2:**
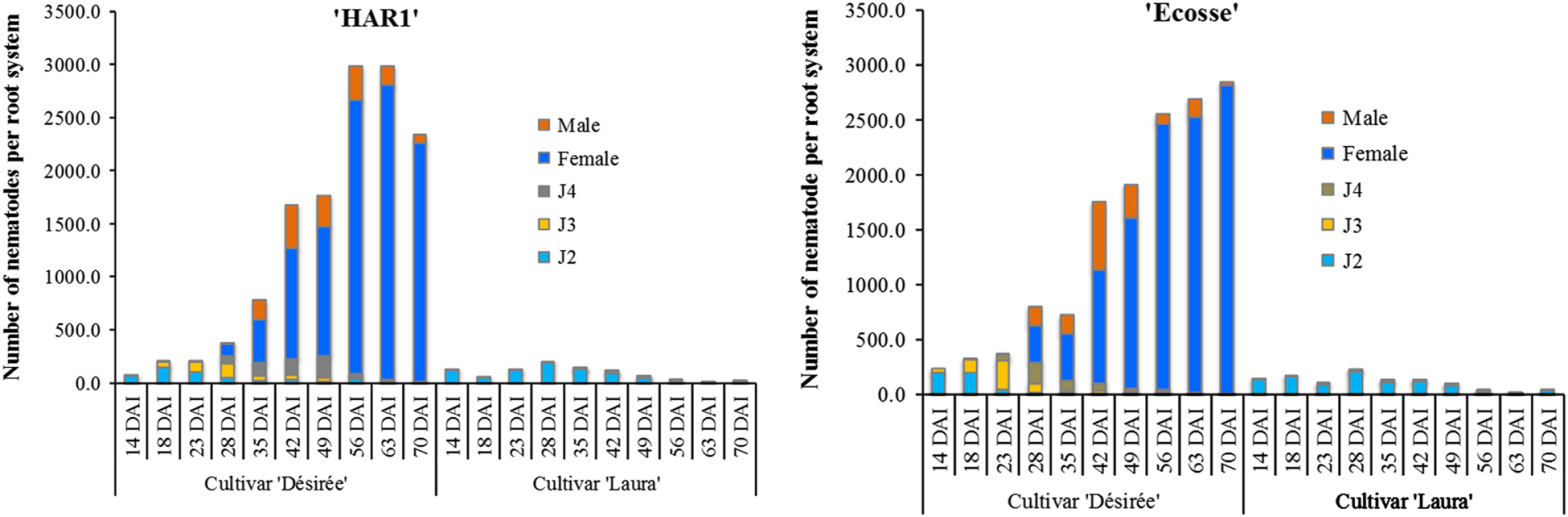
Figure showing the mean number of different developmental stages of *Globodera rostochiensis*, of ‘HAR1’ and ‘Ecosse’ populations in resistant cv. ‘Laura’ and susceptible cv. ‘Désirée’ in the glasshouse in the second experiment.

The number of developing ‘HAR1’ and ‘Ecosse’ *G. rostochiensis* recorded in the roots and soil in the repeat experiment were similar in all treatments. However, some developmental stages were detected earlier than in the first experiment. For instance, at 14 DAI, both J2 and J3 were detected in the roots in all treatments (Fig. 2). At this date, the cumulative degree-days were slightly higher (182 DD_6_) than in the first experiment. Similarly, *G. rostochiensis* J4 were detected five days earlier in experiment two (18 DAI at 239 DD_6_). The first *G. rostochiensis* ‘Ecosse’ females were detected in ‘Désirée’ on 23 DAI at 309 DD_6_, this was five days earlier than in the first experiment while *G. rostochiensis* ‘HAR1’ females were detected five days later (28 DAI at 382 DD_6_). Only males were detected in the roots of ‘Laura’ and in low numbers. The number of days required to complete the life cycle was similar to the first experiment with cumulated DD_6_ of 645 and 736 DD_6_ for ‘Ecosse’ and ‘HAR1’, respectively. The average number of cysts at the end of the experiment was 956 and 876 cysts per root system for *G. rostochiensis* ‘Ecosse’ and ‘HAR1’, respectively.

### Investigating the presence of diapause in Kenya *G. rostochiensis* populations

**Figure 3:**
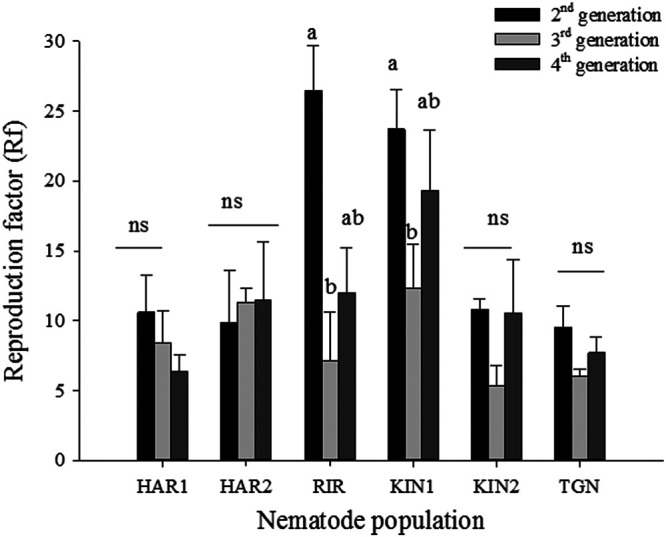
Mean reproduction factor (±SE) of six populations of *Globodera rostochiensis* from Kenya on the susceptible cultivar ‘Désirée’ for three successive generations without diapause. Vertical bars show the standard error of the mean (*n*  = 6). Means within a population with the same small letter are not significantly different (*p* > 0.05).

The *G. rostochiensis* Kenyan populations tested were able to reproduce without diapause (Fig. 3). The Rf values varied significantly among generations for ‘RIR’ and ‘KIN1’, but not the other four populations. In the two former populations, reproduction of the 2nd second generation was higher (*p* < 0.05) than the 3rd generation. The overall reproduction in the 2nd generation ranged from Rf = 9.5 ± 2 in ‘TGN’ to Rf = 26.5 ± 3 in ‘RIR’ (Fig. 3). In the 4th generation, the lowest Rf was recorded for ‘HAR1’ (Rf = 6.4 ± 1) while ‘KIN1’ had the highest (Rf = 19.3 ± 4).

**Figure 4:**
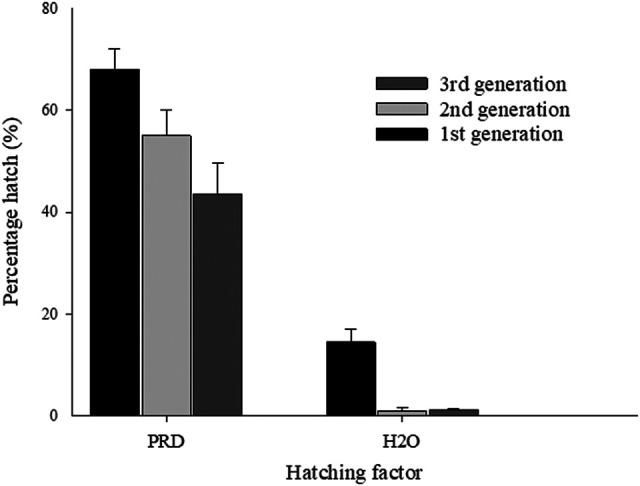
Percentage hatch of *Globodera rostochiensis* ‘HAR1’ population in PRD for three successive generations of cysts without diapause. Vertical bars are mean percentage ± standard error (*n* = 4).

When *G. rostochiensis* ‘HARI’ was incubated in PRD for a period of 10 weeks, up to 68% of the 1st generation cysts hatched, compared to 14% hatch in water. The hatching rate was lower in the subsequent generations with 55 and 44%, respectively (Fig. 4). Hatching in water was less than 2% in the 3rd and the 4th generation cysts.

### Testing the pathotype of *G. rostochiensis* from Kenya

*Globodera rostochiensis* ‘HAR1’ and ‘Ecosse’ had a high Rf on susceptible ‘Désirée’ (Table 1). There was no cyst produced on *S. tuberosum* cv. ‘Laura’ and *S. vernei*, 65.346.19 (Rf = 0), the two clones were therefore resistant to the two populations.

**Table 1.** The reproduction factor (Rf) of *Globodera rostochiensis* populations ‘HAR1’ and ‘Ecosse’ on six differential potato clones^a^.

		Population		Status^c^	
Potato clones	Resistance^b^	‘HAR1’	‘Ecosse’	‘HAR1’	‘Ecosse’
*S. tuberosum* cv. ‘Désirée’	None	61.4	46.6	+	+
*S. tuberosum* cv. ‘Laura’	Ro1,4	0.0	0.0	–	–
*S. kurtzianum*, 60.21.19	Ro1,2	1.1	0.4	+	–
*S. vernei*, 58.1642.4	Ro1,2,3	0.1	0.1	–	–
*S. vernei*, 62.33.3	Ro1,2,3,4	3.2	2.4	+	+
*S. vernei*, 65.346.19	Ro1,2,3,4,5	0.0	0.0	–	–

**Notes:** ^a^The table shows the reproduction factor of ‘HAR1’ population from Kenya and ‘Ecosse’ reference population on six differential potato clones according to Kort et al. (1977). Data are means of two experiments (*n* = *10*); ^b^Ro1, 2, 3, 4, and 5 refers to the five pathotypes of *R. rostochiensis* according to Kort et al. (2077); ^c^(+) indicates a reproduction factor (Rf > 1.0 (susceptible), and (−) indicates a Rf < 1.0 (resistant).

The Rf value on *S. vernei,* 58.1642.4 was <1 for both populations. Equally, both *G. rostochiensis* populations had a low Rf on *S. vernei*, 62.33.3. Only *G. rostochiensis* ‘HAR1’ had a Rf of more than one (Rf = 1.1) on *S. kurtzianum*, 60.21.19 (Table 1).

### Virulence assessment of *G. rostochiensis* from Kenya

The Rf value varied between the *G. rostochiensis* populations ‘Ecosse’, ‘HAR2’, ‘KIN1’, and ‘TGN’. In addition, the populations had a higher Rf in experiment 1 compared to experiment 2 (Table 2). Reproduction of the four populations on in vitro cultivar ‘Désirée’ ranged from an Rf of 14.75 to 37.27 in experiment 1 compared with Rf value of 3.53 to 19.92 in experiment 2. There was no significant difference in Rf among the populations, on ‘Désirée’, except for *G. rostochiensis* ‘HAR2’ in experiment 1 and *G. rostochiensis* ‘KIN1’ in experiment 2, which had significantly lower Rfs (*p* < 0.05) than other populations. All *G. rostochiensis* populations had significantly lower reproduction (*p* < 0.05) on the potato cultivars ‘Rossini’, ‘Caruso’, ‘Amanda’, and ‘Laura’ with an Rf < 1 compared to ‘Désirée’, ‘Connect’, and ‘Performer’ in both experiments (Table 2).

**Table 2.** Response of seven potato cultivars to four populations of *Globodera rostochiensis* under greenhouse conditions.

Cultivar	‘Ecosse’	‘HAR2’	‘KIN1’	‘TGN’
Experiment 1				
‘Désirée’	28.43^d^ ± 12.0^a,A^	14.73 ± 9.6^a,B^	24.97 ± 10.5^a,A^	37.27 ± 22.7^a,A^
‘Connect’	31.70 ± 9.3^a,A^	18.43 ± 5.2^a,B^	30.84 ± 15.3^a,A^	21.80 ± 9.4^a,A^
‘Performer’	2.37 ± 1.3^b,A^	1.74 ± 1.3^bA,B^	2.45 ± 1.9^b,AB^	1.18 ± 0.9^b,B^
‘Rossini’	0.02 ± 0.1^c^	0.00 ± 0.0^c^	0.00 ± 0.0^c^	0.01 ± 0.0^c^
‘Caruso’	0.02 ± 0.1^c^	0.00 ± 0.0^c^	0.00 ± 0.0^c^	0.02 ± 0.1^c^
‘Amanda’	0.00 ± 0.0^c^	0.01 ± 0.0^c^	0.02 ± 0.1^c^	0.05 ± 0.1^c^
‘Laura’^e^	0.00 ± 0.0	0.00 ± 0.0	0.00 ± 0.0	0.00 ± 0.0
Experiment 2				
‘Désirée’	10.07 ± 1.5^a,A^	8.11 ± 1.5^a,A^	3.53 ± 0.3^a,B^	12.92 ± 0.8^a,A^
‘Connect’	7.47 ± 1.1^a,A^	13.31 ± 1.9^a,A^	2.42 ± 0.3^a,B^	12.25 ± 2.7^a,A^
‘Performer’	0.53 ± 0.1^b,AB^	0.35 ± 0.1^b,B^	0.85 ± 0.2^b,A^	1.00 ± 0.3^b,A^
‘Rossini’	0.00 ± 0.0^c^	0.00 ± 0.0^c^	0.04 ± 0.1^c^	0.01 ± 0.0^c^
‘Caruso’	0.00 ± 0.0^c^	0.08 ± 0.3^c^	0.00 ± 0.0^c^	0.02 ± 0.1^c^
‘Amanda’	0.00 ± 0.0^c^	0.00 ± 0.0^c^	0.00 ± 0.0^c^	0.05 ± 0.0^c^
‘Laura’^e^	0.00 ± 0.0	0.00 ± 0.0	0.00 ± 0.0	0.00 ± 0.0

**Notes:** ^d^Reproduction factor (Rf) ± SE. Data are means of two experiments (*n* = *10*). Means of Rf within the same column in each experiment followed with similar lowercase letters are not signiﬁcantly different (*p* > 0.05). Similarly, means of Rf within the same row in each experiment followed with similar capital letters are not significantly different (*p* > 0.05); ^e^Reproduction on cv. ‘Laura’ was tested using eye-plugs due to lack of in vitro plants of this cultivar.

The *G. rostochiensis* population ‘HAR2’ had the lowest reproduction on ‘Connect’ in the first experiment compared to the other *G. rostochiensis* populations with Rf values ranging from 21.8 to 31.7 (Table 2). In the second experiment, all *G. rostochiensis* populations had a similar Rf value on ‘Connect’ except *G. rostochiensis* ‘KIN1’ that was significantly (*p* < 0.05) lower. On ‘Performer’, *G. rostochiensis* populations ‘Ecosse’, ‘HAR1’, and ‘KIN1’ had similar reproduction, while ‘TGN’ had the lowest reproduction in experiment 1. The relative reproduction of all populations on ‘Performer’ was low (Rf ≤ 1) in experiment 2 (Table 2).

Despite the variability in the reproduction of the four populations between the two experiments, the Rs values of the cultivars to the *G. rostochiensis* populations did not differ (Table 3). The potato cultivars, ‘Rossini’, ‘Caruso’, ‘Amanda’, and ‘Laura’ had an Rs value of less than 1% and therefore were ranked as highly resistant (score = 9) according to EPPO (2006). ‘Connect’ was ranked as highly susceptible (score = 1-2) to all populations tested (Table 3). The Rs of ‘Performer’ to the *G. rostochiensis* populations ranged from 5 to 17%. The cultivar had an Rs of 6 for *G. rostochiensis* populations ‘Ecosse’, ‘HAR1’, and ‘TGN’ and a score of 4 for ‘KIN1’ (Table 3).

**Table 3.** Mean relative susceptibility (Rs %) of six potato cultivars to four *G. rostochiensis* populations: ‘Ecosse’, ‘HAR2’, ‘KIN1’, and ‘TGN’^a^.

	‘Ecosse’		‘HAR2’		‘KIN1’		‘TGN’	
Cultivar	Rs (%)^a^	Score	Rs (%)	Score	Rs (%)	Score	Rs (%)	Score
‘Connect’	92.9	2	144.6	1	96.1	2	76.7	2
‘Performer’	6.8	6	8.1	6	17.0	4	5.5	6
‘Rossini’	0.1	9	0.0	9	0.6	9	0.1	9
‘Caruso’	0.1	9	0.5	9	0.0	9	0.2	9
‘Amanda’	0.0	9	0.1	9	0.1	9	0.3	9
‘Laura’^b^	0.0	9	0.0	9	0.0	9	0.0	9

**Notes:** The table shows percentage relative susceptibility (Rs) and the resistance ranking of seven potato cultivars. Data are from two experiments with ten replications per experiment. ^a^Rs (%) = Pf_test cultivar_/Pf_standard susceptible control cultivar_ × 100 and the resistance level (score) determined using the EPPO score scale (1 (>100), 2 (50.1-100%), 3 (25.1-50%), 4 (15.1-25%), 5 (10.1-15%), 6 (5.1-10%), 7 (3.1-5%), 8 (1.1-3%), 9 (≤1%)), where 9 represent the highest level of resistance and 1 represent the least resistant; ^b^Reproduction on cv. ‘Laura’ was tested using eye-plugs due to lack of in vitro plants of this cultivar.

Reproduction of *G. rostochiensis* ‘HAR1’ and ‘Ecosse’ was very low on ‘Albatros’, ‘Belana’, ‘Ribera’, ‘Amado’, ‘Seresta’, and ‘Papageno’ with an Rf < 1 and Rs of less than 1% based on experiments with tuber derived potatoes (Table 4). The six cultivars scored = 9 in resistance. Reproduction of both *G. rostochiensis* populations on ‘Désirée’ was high but not different (*p* > 0.05), although ‘HAR1’ had higher Rf of 56.6 compared to ‘Ecosse’ Rf of 51.1 (Table 4).

**Table 4.** Response of seven potato cultivars to ‘HAR1’ and ‘Ecosse’ populations of *Globodera rostochiensis* under glasshouse conditions^a^.

	‘HAR1’			‘Ecosse’		
Cultivars	Rf ± se	RS (%)	Score	Rf ± se	RS (%)	Score
‘Désirée’	56.56 ± 8.51*	100	1	51.07 ± 4.37*	100	1
‘Albatros’	0.09 ± 0.07	0.16	9	0.02 ± 0.02	0.04	9
‘Belana’	0.10 ± 0.05	0.18	9	0.00 ± 0.00	0.00	9
‘Ribera’	0.08 ± 0.03	0.14	9	0.00 ± 0.00	0.00	9
‘Amado’	0.00 ± 0.00	0.00	9	0.00 ± 0.00	0.00	9
‘Seresta’	0.00 ± 0.00	0.00	9	0.00 ± 0.00	0.00	9
‘Papageno’	0.01 ± 0.02	0.02	9	0.00 ± 0.00	0.00	9

**Note:** ^a^The table shows mean reproduction factor (Rf) ± SE, relative susceptibility (Rs) and respective resistance score of seven potato cultivars. Data are means of two experiments (*n* = 10). Means within the same column and raw with similar asterisk (*) are not signiﬁcantly different (*p* > 0.05).

## Discussion

Potato cyst nematodes have been reported in several regions of Kenya (Mwangi et al., 2015; Mburu et al., 2018, 2020) leading to significant yield losses. To have a better understanding on how to control this nematode, the biology, pathotypes, and virulence of *G. rostochiensis* populations from Kenya were studied in the laboratory and glasshouse.

The resistance mechanism of potato cultivars used in the study did not affect hatching and penetration of the nematode juveniles into the roots over 14 days. As larger cysts of ‘Ecosse’ compared to ‘HAR1’ were used for inoculation, more juveniles penetrated the roots in the first experiment. In the second experiment, only large cysts of the two populations were used, and the number of nematodes of the two populations isolated from the same variety was similar.

Both *G. rostochiensis* ‘HAR1’ and ‘Ecosse’ had similar development pattern in the roots of the resistant potato ‘Laura’. On the resistant cultivar, no females, but males were detected in the roots and the soils. Failure of females to develop in the roots of ‘Laura’ can be attributed to the presence of the H1 resistance gene that confers resistance to pathotypes Ro1 and Ro4 (Bakker et al., 2006; Dalamu et al., 2012). The resistance response involves a hypersensitive reaction leading to necrosis and death of cells surrounding the syncytium (Bakker et al., 2006; Smant et al., 2018). This leaves the developing nematode without enough food and as a result most of them develop into males that do not require a lot of resources (Trudgill, 1991; Moens et al., 2018).

The Kenyan *G. rostochiensis* ‘HAR1’ population required more degree-days (DD_6_) to complete its life cycle compared to the European *G. rostochiensis* population, ‘Ecosse’. The degree-days recorded in our study were higher than what has been reported in other studies (Philis, 1980; Greco et al., 1988; Ebrahimi et al., 2014). The differences in the degree-days resulted from the methods used in the estimation. For example, Philis (1980) calculated the degree-days using the base temperature (minimum heat threshold required by the nematode to develop) of 10°C and the calculation was done from the time of root invasion until the time when eggs containing embryos were noticed. Ebrahimi et al. (2014), on the other hand, used 6°C as the base temperature to calculate DD required for *G. rostochiensis* to complete its life cycle from the J2s of one generation to the J2s of the next generation. In this work, a base temperature of 6°C was used, but the DD were calculated from the day of inoculation (planting day) to the day when the first cyst was detected on the root. However, due to the high temperature and long sampling intervals (5-7 days), there was overlapping of nematode life stages making it difficult to obtain accurate estimates of the DD_6_ required by the populations to complete their life cycle. The final number of cysts was lower than the number of females recorded in the experiment. This indicates that some females did not form eggs or the host roots died before the females could complete the life cycle. However, the data generated in this study indicate that the two nematode populations are a-virulent on ‘Laura’ and that the Kenyan *G. rostochiensis* population ‘HAR1’ requires more DD_6_ than the European reference *G. rostochiensis* population ‘Ecosse’.

The European populations of *G. rostochiensis* have obligatory diapause (Janssen et al., 1987; Muhammad & Evans, 1997; Moens et al., 2018). For that reason, EPPO (2006) standard requires that all populations of PCN used in resistance testing be stored at 4°C for approx. 4 to 6 months prior to the study to break the diapause. The Kenyan populations lacked this obligatory diapause and up to 68% of the newly formed J2 in the eggs were able to hatch immediately and to initiate another generation. This allowed for three successive generations over the short period of nine months. Janssen et al. (1987) described a method of avoiding diapause in PCN which involved crushing newly formed cysts before they undergo desiccation and inoculating them on a host. In this study, the cysts were allowed to undergo complete desiccation and they were not crushed to expose eggs during inoculation. Cysts were wrapped in small retrievable nylon bags, as described above, to distinguish them from newly formed cysts at the end of the experiment to mimic the field situation.

The ability of *G. rostochiensis* to complete more than one generation on the same host has been reported (Philis, 1980; Greco et al., 1988; Jimenez-Perez et al., 2009). The potato used in this study had an early senescence that could not support a second generation on the same host. Nevertheless, this study was designed to simulate the conditions in Kenyan potato production regions, where farmers harvest potatoes and new potato tubers are planted immediately in order to maximize on the number of crop cycles per year.

Potato are grown twice a year in Kenya, but in some areas, farmers are able to grow three potato crops per year by using irrigation (Janssens et al., 2013; Were et al., 2013). Moreover, farmers practice intensive mono cropping of potato with minimum crop rotation (Muthoni et al., 2013) and they allow the proliferation of volunteer potato crops between two potato crop seasons since volunteer potatoes produce early tubers for family consumption. Since *G. rostochiensis* population density in the soil increases with increase in the number of generations per year (Greco, 1993), the aforementioned farm practices could create a conducive environment for multiple generations of nematodes and build-up in the soil thus complicating management.

Both *G. rostochiensis* ‘HAR1’ and ‘Ecosse’ had very high reproduction on ‘Désirée’ which is susceptible to all reported PCN pathotypes. Both of these populations, however, were not able to reproduce on ‘Laura’, *S. vernei* 65.346.19 and *S. vernei* 58.1642.4 (Rf < 1). The *S. vernei*, 65.346.19 clone is resistant to all *G. rostochiensis* pathotypes, but susceptible to *G. pallida* pathotypes (Kort et al., 1977). Failure by *G. rostochiensis* ‘HAR1’ and ‘Ecosse’ to reproduce on this clone confirmed that they were *G. rostochiensis* populations and not a mixture of two species. Cultivar ‘Laura’ carries the H1 gene that confers resistance to Ro1 and Ro4 of *G. rostochiensis* (Bakker et al., 2006). Therefore, it is most likely that the *G. rostochiensis* ‘HAR1’ population belongs to the Ro1/4 pathotype group. Mugniéry et al. (1989) grouped Ro1 and Ro4 together as a-virulent pathotypes on the H1 gene. This was supported by Nijboer and Parlevliet (1990) who found the two pathotypes to have similar virulence patterns.

The pathotyping scheme by Kort et al. (1977) has been criticized particularly for using reproduction factor of >1 or <1 as borderline to separate pathotypes (Trudgill, 1985; Mugniéry et al., 1989; Nijboer and Parlevliet, 1990; da Cunha et al., 2012). This makes it difficult to designate the pathotype in cases when the value is very close to one (Greco et al., 2007). A good example is the reproduction of *G. rostochiensis* ‘HAR1’ on *S. kurtzianum*, 60.21.19 where Rf was 1.1 ± 0.4. Such a small difference may arise from differences in experimental setup leading to the population being designated as the wrong pathotype. However, there is no alternative system available for classification of *G. rostochiensis* populations and the Kort et al. (1977) scheme continues to provide relevant information that is critical in the management of PCN.

Out of 13 cultivars tested, 10 were resistant to Kenyan *G. rostochiensis* populations (Pf < 1), but unfortunately not all the information on the source of resistance of these cultivars is publicly available. The H1 gene has been introgressed into the cultivars ‘Seresta’, ‘Amado’, ‘Amanda’, ‘Caruso’, ‘Rossini’, and ‘Laura’. Like *G. rostochiensis* ‘HAR1’, the Kenyan *G. rostochiensis* populations ‘KIN1’ and ‘TGN’ were not able to reproduce on cultivars having the H1 gene placing them in the Ro1/4 pathotype group. In addition, the Kenyan populations used in this study did not have unusual virulence compared to *G. rostochiensis* ‘Ecosse’ and therefore we concluded that they belong to the same pathotype.

The cultivar ‘Performer’ exhibited partial resistance to the Kenyan populations. The differences in the reproduction of Kenyan *G. rostochiensis* populations on ‘Désirée’, ‘Connect’, and ‘Performer’ between the first and the second experiment might be explained by lack of resistance or the presence of quantitative resistance. Reproduction on such cultivars is strongly influenced by genotype–environment interactions (Trudgill, 1985) and therefore the Rf might differ from one experiment to another.

Information on the pathotype and virulence of a *G. rostochiensis* populations is important in guiding breeding and management programs. The Kenyan populations studied were not able to reproduce on cultivars carrying the H1 gene. Such cultivars can be deployed in the management of *G. rostochiensis*. Most of the potatoes tested were resistant to all the *G. rostochiensis* populations and can therefore be recommended for use by Kenyan growers. There is a need to test more potato cultivars available in Kenya to establish their level of resistance.

Sustainable management of *G. rostochiensis* using the H1 gene should be in the context of integrated pest management, otherwise persistent use of these cultivars imposes selection pressure on the populations. This was reported in the United Kingdom (UK) where continued overuse of potatoes resistant to Ro1, which was the dominant pathotype in the UK, led to the selection and increase of *G. pallida*, which is more difficult to control (Hockland et al., 2012). *G. pallida* was reported in Kenya (Mburu et al., 2018, 2020) at low a frequency and care should be taken to prevent the increase and spread of this species. The use of resistant cultivars tested in this study would significantly decrease nematode accumulation resulting from lack of diapause as reported in this study. *G. rostochiensis* pathotype Ro1/4 seems the prevalent pathotype in Kenya; however, there is a need to test the pathotypes and virulence of more populations before deploying resistant cultivars.
